# Supplementary material for: End Sequence Analysis Toolkit (ESAT) expands the extractable information from single-cell RNA-seq data
Source: Genome Res. 2016 Oct;26(10):1397–410. doi: 10.1101/gr.207902.116 (PMC5052061; doi:10.1101/gr.207902.116)
Supplement: Supplemental Material [file supp_gr.207902.116_Supp_Materials.tar.gz › ESAT-esat_v0.1/doc/html/classumms_1_1core_1_1annotation_1_1_basic_annotation.html]

ESAT: umms.core.annotation.BasicAnnotation Class Reference


ESAT


- Main Page
- Related Pages
- Packages
- Classes
- Files

- Class List
- Class Index
- Class Hierarchy
- Class Members

All Classes Namespaces Files Functions Variables Enumerator Pages

- umms
- core
- annotation
- BasicAnnotation

Classes |
Public Member Functions |
Static Public Member Functions |
Protected Attributes |
List of all members

umms.core.annotation.BasicAnnotation Class Reference

Inheritance diagram for umms.core.annotation.BasicAnnotation:

[legend]

Collaboration diagram for umms.core.annotation.BasicAnnotation:

[legend]

|  |  |
| --- | --- |
| Classes | |
| class | **Factory** |
|  | |

|  |  |
| --- | --- |
| Public Member Functions | |
|  | BasicAnnotation () |
|  | |
|  | BasicAnnotation (SAMRecord samRecord) |
|  | |
|  | BasicAnnotation (String ucsc) |
|  | |
|  | BasicAnnotation (String referenceName, int start, int end, Strand orientation, String name) |
|  | |
|  | BasicAnnotation (String referenceName, Strand orientation, String name, Collection<?extends Annotation > blocks) |
|  | |
|  | BasicAnnotation (String referenceName, int start, int end) |
|  | |
|  | BasicAnnotation (String referenceName, int start, int end, Strand orientation) |
|  | |
|  | BasicAnnotation (String referenceName, int start, int end, String orientation) |
|  | |
|  | BasicAnnotation (BasicAnnotation other) |
|  | |
|  | BasicAnnotation (Annotation other) |
|  | |
|  | BasicAnnotation (Collection<?extends Annotation > blocks) |
|  | |
|  | BasicAnnotation (Collection<?extends Annotation > blocks, Strand orientation, String name) |
|  | |
|  | BasicAnnotation (Collection<?extends Annotation > blocks, String name) |
|  | |
|  | BasicAnnotation (String referenceName, CompoundInterval blocks, Strand orientation, String name) |
|  | |
|  | BasicAnnotation (String referenceName, CompoundInterval blocks, Strand orientation) |
|  | |
|  | BasicAnnotation (String referenceName, CompoundInterval blocks) |
|  | |
| Annotation | copy () |
|  | |
| int | getStart () |
|  | |
| int | getEnd () |
|  | |
| int | getMidpoint () |
|  | |
| String | getReferenceName () |
|  | |
| String | getName () |
|  | |
| Strand | getOrientation () |
|  | |
| boolean | isUnoriented () |
|  | |
| List<?extends Annotation > | getBlocks () |
|  | |
| List<?extends Annotation > | getBlocks (boolean oriented) |
|  | |
| Annotation[] | getFlankingBlocks (Annotation spliceJunction) |
|  | |
| int | numBlocks () |
|  | |
| int | length () |
|  | |
| int | getReferenceCoordinateAtPosition (int positionInAnnotation, boolean ignoreOrientation) |
|  | |
| int | getPositionAtReferenceCoordinate (int referenceCoordinate, boolean ignoreOrientation) |
|  | |
| double | getScore () |
|  | |
| String | toString () |
|  | |
| void | setStart (int start) |
|  | |
| void | setEnd (int end) |
|  | |
| void | setOrientation (Strand orientation) |
|  | |
| void | setOrientedStart (int orientedStart) |
|  | |
| void | setOrientedEnd (int orientedEnd) |
|  | |
| void | setReferenceName (String refName) |
|  | |
| void | setName (String n) |
|  | |
| void | setScore (double s) |
|  | |
| void | addBlocks (Annotation block) |
|  | |
| void | addBlocks (Collection<?extends Annotation > blocks) |
|  | |
| void | shift (int delta) |
|  | |
| void | moveToCoordinate (int coordinateInReference) |
|  | |
| boolean | overlaps (Annotation other, int buffer, boolean considerOrientation) |
|  | |
| boolean | overlaps (List<?extends Annotation > otherBlocks, int buffer) |
|  | |
| int | getOverlap (Annotation other) |
|  | |
| boolean | contains (Annotation other) |
|  | |
| Annotation | union (Annotation other) |
|  | |
| Annotation | intersect (Annotation other) |
|  | |
| List< Annotation > | disect (Annotation a) |
|  | |
| List< Annotation > | disect (List<?extends Annotation > disectors) |
|  | |
| Annotation | minus (Annotation other) |
|  | |
| Annotation | minus (Collection<?extends Annotation > others) |
|  | |
| void | stitchTo (Annotation next) |
|  | |
| Annotation | complement () |
|  | |
| Collection<?extends Annotation > | getSpliceConnections () |
|  | |
| Public Member Functions inherited from umms.core.annotation.AbstractAnnotation | |
| int | size () |
|  | |
| int | getSize () |
|  | |
| int | getMidpoint () |
|  | |
| Strand | getStrand () |
|  | |
| boolean | hasOrientation () |
|  | |
| boolean | isNegativeStrand () |
|  | |
| String | getChr () |
|  | |
| int | getOrientedStart () |
|  | |
| int | getOrientedEnd () |
|  | |
| int | getLengthOnReference () |
|  | |
| int | getReferenceCoordinateAtPosition (int positionInAnnotation) |
|  | |
| int | getPositionAtReferenceCoordinate (int referenceCoordinate) |
|  | |
| void | setOrientation (char orientation) |
|  | |
| void | expand (int deltaStart, int deltaEnd) |
|  | |
| Annotation | trim (int deltaStart, int deltaEnd) |
|  | |
| boolean | fullyContains (Annotation other) |
|  | |
| boolean | overlaps (Annotation other) |
|  | |
| boolean | overlaps (Annotation other, int buffer) |
|  | |
| boolean | overlapsStranded (Annotation other) |
|  | |
| boolean | overlaps (Annotation other, boolean considerOrientation) |
|  | |
| boolean | overlaps (Collection<?extends Annotation > others) |
|  | |
| boolean | overlaps (Collection<?extends Annotation > others, int buffer) |
|  | |
| String | toUCSC () |
|  | |
| String | toBED () |
|  | |
| final String | getFullInfoString () |
|  | |
| String | toBED (int r, int g, int b) |
|  | |
| String | toShortBED () |
|  | |
| String | toBEDGraph () |
|  | |
| int | getDistanceTo (Annotation other) |
|  | |
| String | toCigar () |
|  | |
| List< Annotation > | intersect (List<?extends Annotation > others) |
|  | |
| int | compareTo (Annotation other) |
|  | |
| int | compareToAnnotation (Annotation b) |
|  | |
| int | compareToAnnotation (Annotation b, boolean useOrientation) |
|  | |
| boolean | equals (Annotation a) |
|  | |
| boolean | equals (Annotation a, boolean useOrientation) |
|  | |
| int | getSAMStart () |
|  | |
| int | getSAMEnd () |
|  | |

|  |  |
| --- | --- |
| Static Public Member Functions | |
| static Annotation | createFromUCSC (String ucsc) |
|  | |
| static BasicAnnotation | fromFullInfoString (String fullInfoString) |
|  | |
| static void | main (String[] args) throws IOException |
|  | |
| Static Public Member Functions inherited from umms.core.annotation.AbstractAnnotation | |
| static Strand | getStrand (String orientation) |
|  | |
| static Strand | getStrand (char orientation) |
|  | |

|  |  |
| --- | --- |
| Protected Attributes | |
| CompoundInterval | blocks = new CompoundInterval() |
|  | |

|  |  |
| --- | --- |
| Additional Inherited Members | |
| Static Public Attributes inherited from umms.core.annotation.AbstractAnnotation | |
| static final int | MAX\_DISTANCE = 1000000000 |
|  | |

## Detailed Description

Author
:   engreitz Coordinate system: 0-based, including first base but not the last

## Constructor & Destructor Documentation

|  |  |  |  |  |
| --- | --- | --- | --- | --- |
| umms.core.annotation.BasicAnnotation.BasicAnnotation | ( |  | ) |  |

For Berkeley DB only Do not use this constructor

Here is the caller graph for this function:

|  |  |  |  |  |  |
| --- | --- | --- | --- | --- | --- |
| umms.core.annotation.BasicAnnotation.BasicAnnotation | ( | SAMRecord | *samRecord* | ) |  |

Create an annotation from a sam record The blocks are the mapped blocks This read only; does not consider the mate

Parameters
:   |  |  |
    | --- | --- |
    | samRecord | Annotation with name equal to the read name, orientation and blocks as specified in the alignment |

Here is the call graph for this function:

|  |  |  |  |  |  |
| --- | --- | --- | --- | --- | --- |
| umms.core.annotation.BasicAnnotation.BasicAnnotation | ( | String | *ucsc* | ) |  |

Here is the call graph for this function:

|  |  |  |  |
| --- | --- | --- | --- |
| umms.core.annotation.BasicAnnotation.BasicAnnotation | ( | String | *referenceName*, |
|  |  | int | *start*, |
|  |  | int | *end*, |
|  |  | Strand | *orientation*, |
|  |  | String | *name* |
|  | ) |  |  |

Here is the call graph for this function:

|  |  |  |  |
| --- | --- | --- | --- |
| umms.core.annotation.BasicAnnotation.BasicAnnotation | ( | String | *referenceName*, |
|  |  | Strand | *orientation*, |
|  |  | String | *name*, |
|  |  | Collection<?extends Annotation > | *blocks* |
|  | ) |  |  |

Here is the call graph for this function:

|  |  |  |  |
| --- | --- | --- | --- |
| umms.core.annotation.BasicAnnotation.BasicAnnotation | ( | String | *referenceName*, |
|  |  | int | *start*, |
|  |  | int | *end* |
|  | ) |  |  |

|  |  |  |  |
| --- | --- | --- | --- |
| umms.core.annotation.BasicAnnotation.BasicAnnotation | ( | String | *referenceName*, |
|  |  | int | *start*, |
|  |  | int | *end*, |
|  |  | Strand | *orientation* |
|  | ) |  |  |

|  |  |  |  |
| --- | --- | --- | --- |
| umms.core.annotation.BasicAnnotation.BasicAnnotation | ( | String | *referenceName*, |
|  |  | int | *start*, |
|  |  | int | *end*, |
|  |  | String | *orientation* |
|  | ) |  |  |

|  |  |  |  |  |  |
| --- | --- | --- | --- | --- | --- |
| umms.core.annotation.BasicAnnotation.BasicAnnotation | ( | BasicAnnotation | *other* | ) |  |

Here is the call graph for this function:

|  |  |  |  |  |  |
| --- | --- | --- | --- | --- | --- |
| umms.core.annotation.BasicAnnotation.BasicAnnotation | ( | Annotation | *other* | ) |  |

Here is the call graph for this function:

|  |  |  |  |  |  |
| --- | --- | --- | --- | --- | --- |
| umms.core.annotation.BasicAnnotation.BasicAnnotation | ( | Collection<?extends Annotation > | *blocks* | ) |  |

Here is the call graph for this function:

|  |  |  |  |
| --- | --- | --- | --- |
| umms.core.annotation.BasicAnnotation.BasicAnnotation | ( | Collection<?extends Annotation > | *blocks*, |
|  |  | Strand | *orientation*, |
|  |  | String | *name* |
|  | ) |  |  |

Here is the call graph for this function:

|  |  |  |  |
| --- | --- | --- | --- |
| umms.core.annotation.BasicAnnotation.BasicAnnotation | ( | Collection<?extends Annotation > | *blocks*, |
|  |  | String | *name* |
|  | ) |  |  |

Here is the call graph for this function:

|  |  |  |  |
| --- | --- | --- | --- |
| umms.core.annotation.BasicAnnotation.BasicAnnotation | ( | String | *referenceName*, |
|  |  | CompoundInterval | *blocks*, |
|  |  | Strand | *orientation*, |
|  |  | String | *name* |
|  | ) |  |  |

Here is the call graph for this function:

|  |  |  |  |
| --- | --- | --- | --- |
| umms.core.annotation.BasicAnnotation.BasicAnnotation | ( | String | *referenceName*, |
|  |  | CompoundInterval | *blocks*, |
|  |  | Strand | *orientation* |
|  | ) |  |  |

|  |  |  |  |
| --- | --- | --- | --- |
| umms.core.annotation.BasicAnnotation.BasicAnnotation | ( | String | *referenceName*, |
|  |  | CompoundInterval | *blocks* |
|  | ) |  |  |

## Member Function Documentation

|  |  |  |  |  |  |
| --- | --- | --- | --- | --- | --- |
| void umms.core.annotation.BasicAnnotation.addBlocks | ( | Annotation | *block* | ) |  |

Here is the call graph for this function:

Here is the caller graph for this function:

|  |  |  |  |  |  |
| --- | --- | --- | --- | --- | --- |
| void umms.core.annotation.BasicAnnotation.addBlocks | ( | Collection<?extends Annotation > | *blocks* | ) |  |

Here is the call graph for this function:

|  |  |  |  |  |
| --- | --- | --- | --- | --- |
| Annotation umms.core.annotation.BasicAnnotation.complement | ( |  | ) |  |

Returns
:   a new CompoundInterval containing the gaps between the blocks in this annotation

Implements umms.core.annotation.Annotation.

Here is the call graph for this function:

|  |  |  |  |  |  |
| --- | --- | --- | --- | --- | --- |
| boolean umms.core.annotation.BasicAnnotation.contains | ( | Annotation | *other* | ) |  |

Parameters
:   |  |  |
    | --- | --- |
    | other |  |

Returns
:   true if the blocks of other are fully contained within the blocks of this object

Implements umms.core.annotation.Annotation.

Here is the call graph for this function:

|  |  |  |  |  |
| --- | --- | --- | --- | --- |
| Annotation umms.core.annotation.BasicAnnotation.copy | ( |  | ) |  |

Returns
:   A deep clone of this Annotation object

Implements umms.core.annotation.Annotation.

Here is the call graph for this function:

|  |  |  |  |  |  |
| --- | --- | --- | --- | --- | --- |
| static Annotation umms.core.annotation.BasicAnnotation.createFromUCSC | ( | String | *ucsc* | ) |  |

static

Here is the call graph for this function:

|  |  |  |  |  |  |
| --- | --- | --- | --- | --- | --- |
| List<Annotation> umms.core.annotation.BasicAnnotation.disect | ( | Annotation | *a* | ) |  |

Fragments the current annotation if it overlaps the provided one. it returns an empty list if this annotation does not overlap the one passed in.

Parameters
:   |  |  |
    | --- | --- |
    | Annotation | annotation to disect the current one |

Returns
:   List<Annotation> of the disected annotations, an empty list is returned if the annotations do not overlap.

Implements umms.core.annotation.Annotation.

Here is the caller graph for this function:

|  |  |  |  |  |  |
| --- | --- | --- | --- | --- | --- |
| List<Annotation> umms.core.annotation.BasicAnnotation.disect | ( | List<?extends Annotation > | *disectors* | ) |  |

Fragments the current annotation if it overlaps the provided ones. It returns a list with one component (this annotation) if no annotation in the provided list overlaps the discted annotaion.

Parameters
:   |  |  |
    | --- | --- |
    | List<GenomicAnnotation> | **sorted** annotations with which to disect the current one. |

Returns
:   List<GenomicAnnotation> of the disected annotations, a list just this annotation is returned if the annotations do not overlap.

Implements umms.core.annotation.Annotation.

Here is the call graph for this function:

|  |  |  |  |  |  |
| --- | --- | --- | --- | --- | --- |
| static BasicAnnotation umms.core.annotation.BasicAnnotation.fromFullInfoString | ( | String | *fullInfoString* | ) |  |

static

Create from string produced by AbstractAnnotation.getFullInfoString();

Parameters
:   |  |  |
    | --- | --- |
    | fullInfoString | Full info string |

Returns
:   Corresponding annotation object

Here is the call graph for this function:

|  |  |  |  |  |
| --- | --- | --- | --- | --- |
| List<? extends Annotation> umms.core.annotation.BasicAnnotation.getBlocks | ( |  | ) |  |

Returns unoriented blocks for this gene

Implements umms.core.annotation.Annotation.

Here is the caller graph for this function:

|  |  |  |  |  |  |
| --- | --- | --- | --- | --- | --- |
| List<? extends Annotation> umms.core.annotation.BasicAnnotation.getBlocks | ( | boolean | *oriented* | ) |  |

Parameters
:   |  |  |
    | --- | --- |
    | oriented | set to true if the list should be ordered according to the annotation orientation and by position |

Returns

Implements umms.core.annotation.Annotation.

Here is the call graph for this function:

|  |  |  |  |  |
| --- | --- | --- | --- | --- |
| int umms.core.annotation.BasicAnnotation.getEnd | ( |  | ) |  |

Returns
:   end of the annotation (exclusive)

Implements umms.core.annotation.Annotation.

Here is the caller graph for this function:

|  |  |  |  |  |  |
| --- | --- | --- | --- | --- | --- |
| Annotation [] umms.core.annotation.BasicAnnotation.getFlankingBlocks | ( | Annotation | *spliceJunction* | ) |  |

This function returns the blocks on either ends of a given splicejunction

Parameters
:   |  |  |
    | --- | --- |
    | spliceJunction |  |

Returns

Here is the call graph for this function:

|  |  |  |  |  |
| --- | --- | --- | --- | --- |
| int umms.core.annotation.BasicAnnotation.getMidpoint | ( |  | ) |  |

This will return the position in the center of this annotation taking into account the blocked structure

Returns

Implements umms.core.annotation.Annotation.

Here is the call graph for this function:

|  |  |  |  |  |
| --- | --- | --- | --- | --- |
| String umms.core.annotation.BasicAnnotation.getName | ( |  | ) |  |

Returns
:   the name of the annotation

Implements umms.core.annotation.Annotation.

Here is the caller graph for this function:

|  |  |  |  |  |
| --- | --- | --- | --- | --- |
| Strand umms.core.annotation.BasicAnnotation.getOrientation | ( |  | ) |  |

Returns
:   orientation or strand

Implements umms.core.annotation.Annotation.

Here is the caller graph for this function:

|  |  |  |  |  |  |
| --- | --- | --- | --- | --- | --- |
| int umms.core.annotation.BasicAnnotation.getOverlap | ( | Annotation | *other* | ) |  |

Returns the number of bases that overlap between the two annotations.

Parameters
:   |  |  |
    | --- | --- |
    | other |  |

Returns

Implements umms.core.annotation.Annotation.

Here is the call graph for this function:

|  |  |  |  |
| --- | --- | --- | --- |
| int umms.core.annotation.BasicAnnotation.getPositionAtReferenceCoordinate | ( | int | *referenceCoordinate*, |
|  |  | boolean | *ignoreOrientation* |
|  | ) |  |  |

Implements umms.core.annotation.Annotation.

Here is the call graph for this function:

Here is the caller graph for this function:

|  |  |  |  |
| --- | --- | --- | --- |
| int umms.core.annotation.BasicAnnotation.getReferenceCoordinateAtPosition | ( | int | *positionInAnnotation*, |
|  |  | boolean | *ignoreOrientation* |
|  | ) |  |  |

Implements umms.core.annotation.Annotation.

Here is the call graph for this function:

Here is the caller graph for this function:

|  |  |  |  |  |
| --- | --- | --- | --- | --- |
| String umms.core.annotation.BasicAnnotation.getReferenceName | ( |  | ) |  |

Returns
:   name of the reference sequence

Implements umms.core.annotation.Annotation.

Here is the caller graph for this function:

|  |  |  |  |  |
| --- | --- | --- | --- | --- |
| double umms.core.annotation.BasicAnnotation.getScore | ( |  | ) |  |

Implements umms.core.annotation.Annotation.

Here is the caller graph for this function:

|  |  |  |  |  |
| --- | --- | --- | --- | --- |
| Collection<? extends Annotation> umms.core.annotation.BasicAnnotation.getSpliceConnections | ( |  | ) |  |

The BasicAnnotation will return the gaps themselves

Implements umms.core.annotation.Annotation.

Here is the call graph for this function:

Here is the caller graph for this function:

|  |  |  |  |  |
| --- | --- | --- | --- | --- |
| int umms.core.annotation.BasicAnnotation.getStart | ( |  | ) |  |

Returns
:   start of the annotation (inclusive)

Implements umms.core.annotation.Annotation.

Here is the caller graph for this function:

|  |  |  |  |  |  |
| --- | --- | --- | --- | --- | --- |
| Annotation umms.core.annotation.BasicAnnotation.intersect | ( | Annotation | *other* | ) |  |

Returns the result of intersecting this instance with another

Parameters
:   |  |  |
    | --- | --- |
    | other |  |

Returns
:   returns null if there is no intersection

Implements umms.core.annotation.Annotation.

Here is the call graph for this function:

|  |  |  |  |  |
| --- | --- | --- | --- | --- |
| boolean umms.core.annotation.BasicAnnotation.isUnoriented | ( |  | ) |  |

Returns
:   false if the annotation is not in either negative or positive orientation

Implements umms.core.annotation.Annotation.

Here is the call graph for this function:

|  |  |  |  |  |
| --- | --- | --- | --- | --- |
| int umms.core.annotation.BasicAnnotation.length | ( |  | ) |  |

Returns
:   length of the Annotation; if it has blocks, the gaps between blocks are not included

Implements umms.core.annotation.Annotation.

Here is the caller graph for this function:

|  |  |  |  |  |  |
| --- | --- | --- | --- | --- | --- |
| static void umms.core.annotation.BasicAnnotation.main | ( | String[] | *args* | ) | throws IOException |

static

Here is the call graph for this function:

|  |  |  |  |  |  |
| --- | --- | --- | --- | --- | --- |
| Annotation umms.core.annotation.BasicAnnotation.minus | ( | Annotation | *other* | ) |  |

Returns the difference (all regions in this genomic annotation not in the given other annotation) between this genomic annotation and the one

Parameters
:   |  |  |
    | --- | --- |
    | other | - the annotations to take the difference against |

Returns
:   A blocked annotation of the resulting difference.

Implements umms.core.annotation.Annotation.

Here is the call graph for this function:

Here is the caller graph for this function:

|  |  |  |  |  |  |
| --- | --- | --- | --- | --- | --- |
| Annotation umms.core.annotation.BasicAnnotation.minus | ( | Collection<?extends Annotation > | *others* | ) |  |

Returns the difference (all regions in this genomic annotation not in the given list) between this genomic annotation and the given list

Parameters
:   |  |  |
    | --- | --- |
    | others | - the annotations to take the difference against |

Returns
:   A blocked annotation of the resulting difference.

Implements umms.core.annotation.Annotation.

Here is the call graph for this function:

|  |  |  |  |  |  |
| --- | --- | --- | --- | --- | --- |
| void umms.core.annotation.BasicAnnotation.moveToCoordinate | ( | int | *coordinateInReference* | ) |  |

Move an annotation, preserving the relationships between its blocks, to a new coordinate.

Parameters
:   |  |  |
    | --- | --- |
    | coordinateInReference |  |

Implements umms.core.annotation.Annotation.

|  |  |  |  |  |
| --- | --- | --- | --- | --- |
| int umms.core.annotation.BasicAnnotation.numBlocks | ( |  | ) |  |

Returns
:   number of blocks in the Annotation

Implements umms.core.annotation.Annotation.

Here is the caller graph for this function:

|  |  |  |  |
| --- | --- | --- | --- |
| boolean umms.core.annotation.BasicAnnotation.overlaps | ( | Annotation | *other*, |
|  |  | int | *buffer*, |
|  |  | boolean | *considerOrientation* |
|  | ) |  |  |

Implements umms.core.annotation.Annotation.

Here is the call graph for this function:

Here is the caller graph for this function:

|  |  |  |  |
| --- | --- | --- | --- |
| boolean umms.core.annotation.BasicAnnotation.overlaps | ( | List<?extends Annotation > | *otherBlocks*, |
|  |  | int | *buffer* |
|  | ) |  |  |

Here is the call graph for this function:

|  |  |  |  |  |  |
| --- | --- | --- | --- | --- | --- |
| void umms.core.annotation.BasicAnnotation.setEnd | ( | int | *end* | ) |  |

Sets a new end position. Will delete and/or truncate blocks if the new end position is less than the old one. Will extend the last block if the new end position is greater than old one.

Parameters
:   |  |  |
    | --- | --- |
    | new | end position (ignoring orientation) of the annotation |

Implements umms.core.annotation.Annotation.

Here is the caller graph for this function:

|  |  |  |  |  |  |
| --- | --- | --- | --- | --- | --- |
| void umms.core.annotation.BasicAnnotation.setName | ( | String | *name* | ) |  |

Parameters
:   |  |  |
    | --- | --- |
    | name | name of the annotation |

Implements umms.core.annotation.Annotation.

Here is the caller graph for this function:

|  |  |  |  |  |  |
| --- | --- | --- | --- | --- | --- |
| void umms.core.annotation.BasicAnnotation.setOrientation | ( | Strand | *orientation* | ) |  |

Implements umms.core.annotation.Annotation.

Here is the caller graph for this function:

|  |  |  |  |  |  |
| --- | --- | --- | --- | --- | --- |
| void umms.core.annotation.BasicAnnotation.setOrientedEnd | ( | int | *orientedEnd* | ) |  |

Implements umms.core.annotation.Annotation.

Here is the call graph for this function:

|  |  |  |  |  |  |
| --- | --- | --- | --- | --- | --- |
| void umms.core.annotation.BasicAnnotation.setOrientedStart | ( | int | *orientedStart* | ) |  |

Implements umms.core.annotation.Annotation.

Here is the call graph for this function:

|  |  |  |  |  |  |
| --- | --- | --- | --- | --- | --- |
| void umms.core.annotation.BasicAnnotation.setReferenceName | ( | String | *refName* | ) |  |

Parameters
:   |  |  |
    | --- | --- |
    | refName | name of the reference sequence / chromosome |

Implements umms.core.annotation.Annotation.

Here is the caller graph for this function:

|  |  |  |  |  |  |
| --- | --- | --- | --- | --- | --- |
| void umms.core.annotation.BasicAnnotation.setScore | ( | double | *s* | ) |  |

Implements umms.core.annotation.Annotation.

Here is the caller graph for this function:

|  |  |  |  |  |  |
| --- | --- | --- | --- | --- | --- |
| void umms.core.annotation.BasicAnnotation.setStart | ( | int | *start* | ) |  |

Sets a new start position. Will delete and/or truncate blocks if the new start position is greater than the old one. Will extend the first block if the new start position is less than old one.

Parameters
:   |  |  |
    | --- | --- |
    | new | start position (ignoring orientation) of the annotation |

Implements umms.core.annotation.Annotation.

Here is the caller graph for this function:

|  |  |  |  |  |  |
| --- | --- | --- | --- | --- | --- |
| void umms.core.annotation.BasicAnnotation.shift | ( | int | *delta* | ) |  |

Shift the positioning of the annotation by a given number of base pairs.

Parameters
:   |  |  |
    | --- | --- |
    | delta | Positive or negative shift to apply to all blocks in the annotation. delta=0  leaves the annotation unchanged. |

Implements umms.core.annotation.Annotation.

|  |  |  |  |  |  |
| --- | --- | --- | --- | --- | --- |
| void umms.core.annotation.BasicAnnotation.stitchTo | ( | Annotation | *next* | ) |  |

Implements umms.core.annotation.Annotation.

Here is the call graph for this function:

|  |  |  |  |  |
| --- | --- | --- | --- | --- |
| String umms.core.annotation.BasicAnnotation.toString | ( |  | ) |  |

Here is the call graph for this function:

|  |  |  |  |  |  |
| --- | --- | --- | --- | --- | --- |
| Annotation umms.core.annotation.BasicAnnotation.union | ( | Annotation | *other* | ) |  |

Parameters
:   |  |  |
    | --- | --- |
    | other |  |

Returns
:   union of current instance and other object. If strands agree, then strand is set to that. If the strands conflict, strand is set to UNKNOWN

Implements umms.core.annotation.Annotation.

Here is the call graph for this function:

## Member Data Documentation

CompoundInterval umms.core.annotation.BasicAnnotation.blocks = new CompoundInterval()

protected

---

The documentation for this class was generated from the following file:

- java/umms/core/annotation/BasicAnnotation.java


---

Generated on Fri Aug 15 2014 15:11:08 for ESAT by  

 1.8.7
